# Radiomics Features from Different Prostatic Zones on ^18^F-PSMA-1007 PET/CT for Predicting Persistent PSA in Prostate Cancer Patients: A Multicenter Study

**DOI:** 10.3390/cancers17172807

**Published:** 2025-08-28

**Authors:** Licong Li, Jian Xu, Shuying Bian, Fei Yao, Qi Lin, Meiyan Zhou, Yunjun Yang, Meiyao Song, Yixuan Pan, Qinyang Shen, Yuandi Zhuang, Jie Lin

**Affiliations:** 1 The Department of Radiology, The First Affiliated Hospital of Wenzhou Medical University, Wenzhou 325000, Chinazhuangyuandi@wmu.edu.cn (Y.Z.); 2 The Department of Urology, The First Affiliated Hospital of Wenzhou Medical University, Wenzhou 325000, China; 3 The Department of Radiology, The People’s Hospital of Yuhuan, Yuhuan 317600, China; 4 The Department of Nuclear Medicine, The First Affiliated Hospital of Wenzhou Medical University, Wenzhou 325000, China; 5 The First School of Clinical Medicine (School of Information and Engineering), Wenzhou Medical University, Wenzhou 325000, China

**Keywords:** radiomics, persistent PSA, tumor microenvironment, prostate cancer, PET/CT

## Abstract

**Objectives**: This study aims to explore the role of radiomics features (RFs) from prostate subregions, including the tumor microenvironment (TME), in predicting persistent PSA. **Methods**: In retrospective analysis, we segregated 354 patients with pathologically confirmed localized prostate cancer (PCa) into training, internal validation, and external validation cohorts. The prostate on ^18^F-prostate-specific membrane antigen (PSMA)-1007 positron emission tomography/computed tomography (PET/CT) was partitioned into three zones based on the maximum standardized uptake value (SUVmax) (zone-intra: 45–100% SUVmax; zone-peri: 20–45% SUVmax; zone-norm: 0–20% SUVmax). RFs from these zones were harnessed to develop five radiomics models [model-intra; model-peri; model-norm; model-ip; model-ipn]. Three optimal radiomics models were further integrated with the PSA model to construct combined models. Model performance was evaluated using the receiver operating characteristic (ROC) curves and the area under the curve (AUC). **Results**: Utilizing least absolute shrinkage and selection operator (LASSO) and logistic regression, five radiomics models were constructed, with model-ip, model-ipn, and model-intra showing superior performance [training cohort AUCs: 0.76 (0.68–0.83), 0.75 (0.68–0.83), 0.76 (0.68–0.83); internal validation cohort AUCs: 0.76 (0.65–0.88), 0.72 (0.57–0.86), 0.70 (0.55–0.86); external validation cohort AUCs: 0.70 (0.50–0.86), 0.55 (0.36–0.73), 0.53 (0.34–0.72)]. Notably, the combined model incorporating model-ip and the PSA model exhibited optimal performance [training cohort AUC: 0.78 (0.71–0.85); internal validation cohort AUC: 0.78 (0.67–0.90); external validation cohort AUC: 0.89 (0.72–0.98)]. **Conclusions**: The RFs in different subregions on ^18^F-PSMA-1007 PET/CT have varying effectiveness in predicting persistent PSA. A radiomics model that encompasses the 20–45% SUVmax and 45–100% SUVmax zones, when combined with the PSA model, enhances predictive accuracy.

## Simple Summary

Persistent prostate-specific antigen (PSA) following radical prostatectomy in localized prostate cancer indicates adverse prognosis. Utilizing real-world data from 354 patients, this study developed a preoperative combined model incorporating tumor microenvironment radiomics features to predict persistent PSA. This approach enhances predictive accuracy and may facilitate early therapeutic intervention in high-risk patients.

## 1. Introduction

Prostate cancer (PCa) is one of the most prevalent malignancies among male patients, with a continuously increasing incidence in recent years in China [1]. Prostate-specific antigen (PSA) is a routine indicator used for early-stage PCa screening [2,3]. In addition to preliminary screening, PSA can also be used to assess the prognosis of patients after radical prostatectomy (RP). Persistent PSA, which is defined as PSA ≥ 0.1 ng/mL within 4–8 weeks after RP, is considered to be associated with worse tumor outcomes such as cancer progression, biochemical recurrence, and cancer-specific mortality, leading to a worse prognosis if interventions are delayed [4,5,6,7,8].

Prostate-specific membrane antigen (PSMA) positron emission tomography/computed tomography (PET/CT) is another commonly used method for accurate diagnosis of PCa recurrence and metastasis [9]. PSMA is a transmembrane protein that is weakly expressed in prostate tissue and usually increases rapidly during cancer development. It can be bound with ligands for the diagnosis and treatment of PCa [10]. PET/CT is a multimodality imaging technique that combines functional and morphological information, enabling accurate identification and localization of PSMA bound to ligands labeled with radioactive markers [11]. However, even when persistent PSA is present, the disease may not manifest with extensive recurrence and metastasis, making visual detection of PSMA-targeted imaging with PET/CT challenging.

The advent of radiomics has broken through this limitation. It uses a computer to recognize microscopic radiomics features (RFs) that are invisible to the naked eye, providing a new perspective for PCa diagnosis. Radiomics, a rapidly developing field, involves the extraction of a multitude of quantitative RFs within the region of interest. These RFs capture tissue and lesion characteristics, providing complementary information about tumor heterogeneity within the whole tumor volume, which improves survival prediction and supports clinical decisions [12]. Many studies have shown that RFs could be used for early screening and prognosis prediction of PCa [13,14,15].

Recently, with the deepening of research, the focus of radiomics has been expanding beyond the core of the lesion to the tumor periphery, which is called the tumor microenvironment (TME) [13,16,17,18]. It is an environment that arises under the interaction between tumor cells and host cells to promote tumor growth and metastasis. Due to the different tumor types, the TME could contain different cells or non-cellular components, and the non-immune matrix population of PCa is an important part of the TME, showing high endothelial angiogenic activity in the PCa microenvironment, which affects tumor progression [19,20,21,22].

Based on the understanding that TME can affect tumor progression, our study defined TME by the maximum standardized uptake value (SUVmax) on preoperative ^18^F-PSMA-1007 PET/CT images and further explored the predictive performance of RFs in different subregions, including TME on persistent PSA to judge the prognosis of tumors. Concurrently, to stratify patient risks before surgery for prognostic management (more targeted treatment of high-risk patients, improved social benefits, and facilitating patient follow-up), we incorporate preoperative clinical parameters for prediction and comparison.

## 2. Materials and Methods

### 2.1. Patient Selection

**Figure 1:**
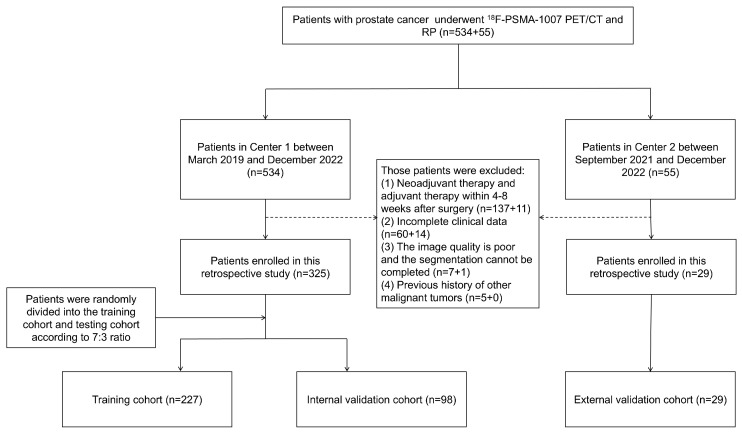
The patient selection procedure.

This study has been approved by the Ethics Review Committee of the First Affiliated Hospital of Wenzhou Medical University (Center 1) and the First Affiliated Hospital of Zhejiang University (Center 2). All patients included have signed the informed consent form. This retrospective study included 354 patients with pathologically confirmed PCa from Center 1 between March 2019 and December 2022 and Center 2 between September 2019 and December 2022. All patients underwent a standardized preoperative ^18^F-PSMA-1007 PET/CT examination and RP. We collected the clinical information, PSA follow-up data, and PSMA PET/CT images of all patients. Exclusion criteria were as follows: (1) neoadjuvant therapy and adjuvant therapy within 4–8 weeks after surgery; (2) incomplete clinical data; (3) poor image quality, leading to failure in completing segmentation; and (4) previous history of other malignant tumors. Patients from Center 1 were randomly divided into the training cohort and the internal validation cohort in a 7:3 ratio. The external validation cohort was formed by patients from Center 2. The details are shown in Figure 1.

### 2.2. ^18^F-PSMA-1007 PET/CT Image Acquisition and ROI Segmentation

^18^F-PSMA-1007 was administered by a slow intravenous injection of 4.0 MBq/kg (median activity: 282.7 MBq; range: 170.2–366.3 MBq), and the scan was performed 120 min after injection. The whole-body examination (from the top of the head to the mid-thighs) was performed using a low-dose CT scan with the following parameters (140 Kvp, 110 mA): detector collimation of 64 × 0.625 mm, pitch of 0.829, a tube rotation speed of 0.5 s, section thickness of 5 mm, and reconstruction thickness of 2.5 mm. This was followed by a PET scan of the same area that matched the CT section thickness. All scans were acquired on a hybrid PET/CT scanner (Gemini TF 64, Philips, Best, The Netherlands). All PET scans were acquired in 3D mode with an acquisition time of 1.5 min per bed position, and the overlap between two adjacent bed positions was 50%. The parameters of the PET image are as follows: field of view, 576 mm; matrix of 144 × 144; and slice thickness and interval, 5 mm. PET images with CT attenuation correction were reconstructed using the time-of-flight algorithm.

**Figure 2:**
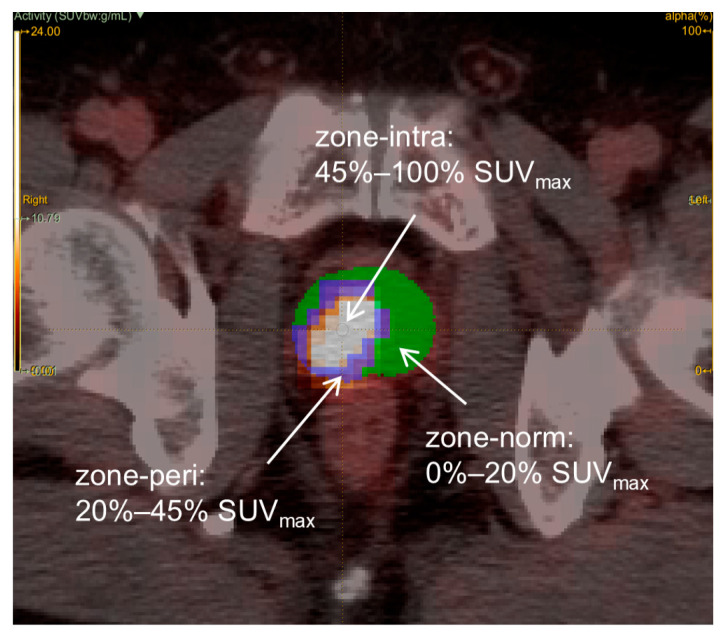
The different subregions delineated of the prostate on ^18^F-PSMA-1007 PET/CT.

After aligning the PET and CT images of patients, we imported the PET/CT images of all patients into the LIFEx software (version 7.3; https://www.lifexsoft.org, accessed on 13 December 2023) [23]. PET images were resampled to have voxel dimensions of 2*2*2 mm^3^. The entire prostate was semiautomatically divided as a region of interest (ROI) on the ^18^F-PSMA-1007 PET/CT images by two nuclear medicine physicians with more than 5 years of work experience. Neither of these physicians knew any clinical information about the patients. In existing studies, the delineation of PCa lesions is commonly performed using a threshold of 40–50% SUVmax, a method validated and widely adopted [24]. Thus, we defined the intratumoral region using a threshold of 45% SUVmax. In contrast, defining the TME requires a broader boundary to encompass the areas of interaction between tumor cells and host cells. We selected the 20% SUVmax threshold as the boundary for the TME primarily because Spohn et al. demonstrated that tumor volumes delineated by semiautomatic segmentation using the 20% SUVmax threshold showed no significant difference compared to histopathological reference volumes and exhibited high sensitivity and specificity [25]. Furthermore, this threshold effectively balances lesion coverage with the protection of normal tissue, thereby minimizing both over-segmentation and the omission of invasive tumor margins. This approach likely encompasses key regions involved in the dynamic interaction between the tumor and its stroma. Based on this, the prostate was delineated by the software based on the SUVmax threshold, which was defined as 45–100% SUVmax, 20–45% SUVmax, and 0–20% SUVmax. The zone-intra (45–100% SUVmax) exhibits high tracer uptake, representing the metabolically active, rapidly proliferating tumor core. In contrast, the zone-norm (0–20% SUVmax) demonstrates low radioactivity, corresponding to normal prostate tissue devoid of tumor invasion. The zone-peri (20–45% SUVmax) displays SUV values intermediate between the zone-intra and zone-norm, indicative of the tumor–normal prostate interface and representing the TME [26,27], as illustrated in Figure 2. It is worth noting that physiological uptake outside the prostate and urethral residues are excluded.

### 2.3. Feature Extraction and Selection

**Figure 3:**
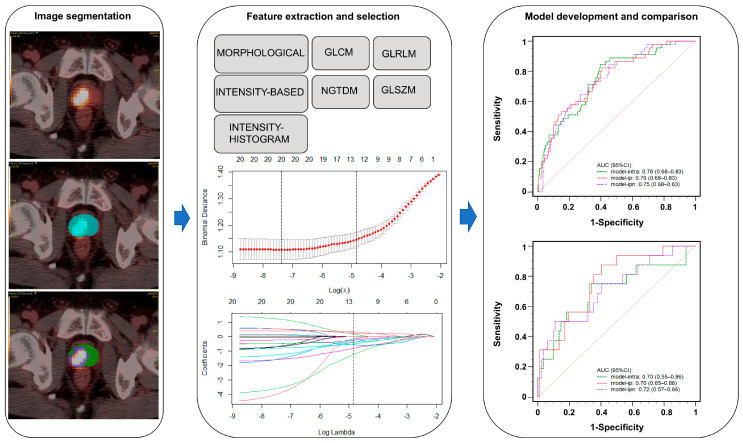
The radiomics flowchart.

We used LIFEx software to extract a comprehensive set of 125 RFs from each delineated subregion on PET images (RFs: MORPHOLOGICAL, INTENSITY-BASED, INTENSITY-HISTOGRAM, GLCM, GLRLM, NGTDM, and GLSZM). The consistency of the RFs extracted by the two physicians was assessed by the intra-class correlation coefficient (ICC). If the ICC > 0.75, the extracted features were considered to have high consistency and high stability. All the features were normalized with the z-score. In order to address the unbalanced classification, we used synthetic minority over-sampling in the training cohort to increase the sample size. Then, the 20 standardized features taken by each model were selected by the maximum relevance minimum redundancy (mRMR), and the resulting features were further filtered by least absolute shrinkage and selection operator (LASSO). The radiomics flowchart is shown in Figure 3.

### 2.4. Establishment and Testing of the Models

**Figure 4:**
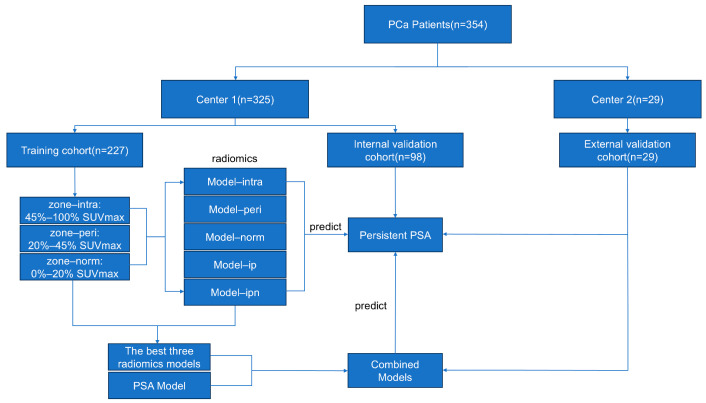
The flowchart of study.

The flowchart of the study is shown in Figure 4. Through logistic regression, we developed five radiomics models incorporating distinct zones based on SUVmax threshold ranges (model-intra: 45–100% SUVmax; model-peri: 20–45% SUVmax; model-norm: 0–20% SUVmax; model-ip: 20–45% SUVmax + 45–100% SUVmax; model-ipn: 0–20% SUVmax + 20–45% SUVmax + 45–100% SUVmax). Finally, the rad-scores were calculated from filtered RFs. These rad-scores formed the basis of all radiomics models. In the training cohort, we used univariate and multifactorial logistic regression to obtain the independent predictors, including rad-score and serum total prostate-specific antigen (tPSA). Then, we built the radiomics models with rad-score, set up the PSA model with tPSA values, and combined the three best radiomics models with the PSA model. Internal and external validation cohorts were used to test models for their performance. Persistent PSA was defined as PSA ≥ 0.1 ng/mL within 4–8 weeks after RP in patients with PCa.

### 2.5. Statistical Analysis

All analyses in this study were performed using the R software (version 4.3.1; https://www.r-project.org, accessed on 8 July 2023). In the statistical description, categorical variables were described by count and percentage, continuous variables that meet normality were described by mean ± standard deviation, and continuous variables that do not satisfy normality were expressed as medians with interquartile ranges. The Mann–Whitney U test was used to test the difference in the International Society of Urological Pathology (ISUP) grade between different cohorts, and the Analysis of Variance or the Mann–Whitney U test was used to analyze the differences between continuous variables. The sensitivity, specificity, and the area under the curve (AUC) were used to evaluate the performance of the models. The statistical significance of the difference in AUC between different models was tested by using the DeLong test. The net reclassification index (NRI) and integrated discrimination improvement (IDI) were used to assess the clinical benefit and utility of different models. The decision curve analysis (DCA) was performed to assess the clinical utility of the model using the ‘rmda’ package. *p* < 0.05 was set up as statistically significant.

## 3. Results

### 3.1. Patient Characteristics

The 354 patients included were randomly divided into the training cohort (*n* = 227), the internal validation cohort (*n* = 98), and the external validation cohort (*n* = 29). The baseline characteristics of three cohorts are shown in Table 1.

### 3.2. The Construction of Different Models

In the training cohort, we used univariate logistic regression of age, height, weight, body mass index (BMI), tPSA, free prostate-specific antigen (fPSA), and tumor SUVmax to yield independent predictors of persistent PSA. Finally, the tPSA was obtained to construct the PSA model (*p* < 0.05). Furthermore, we obtained 164 RFs extracted from different zones of the PET images, and 125 RFs were obtained after excluding the RFs that could not be used for statistical analysis. Of these, RFs with ICC > 0.75 were summarized to obtain 88 RFs in zone-intra, 119 RFs in zone-peri, and 124 RFs in zone-norm. The models were constructed using the final RFs obtained in each zone. It is worth noting that several common RFs are visible in the three most superior radiomics models such as GLSZM. More details are shown in Supplementary Material Table S1.

### 3.3. The Performance of Different Models

Table 2 shows the AUC, specificity, and sensitivity of models. The three most superior radiomics models were model-ip, model-ipn, and model-intra. Their AUCs (95%CI) were 0.76 (0.68–0.83), 0.75 (0.68–0.83), and 0.76 (0.68–0.83) in the training cohort, 0.76 (0.65–0.88), 0.72 (0.57–0.86), and 0.70 (0.55–0.86) in the internal validation cohort, and 0.70 (0.50–0.86), 0.55 (0.36–0.73), and 0.53 (0.34–0.72) in the external validation cohort. In the training cohort, the AUC (95%CI) of the PSA model was 0.74 (0.65–0.82), while in the validation cohort, it was 0.68 (0.54–0.82) and 0.88 (0.71–0.97), both internal cohorts lower than the three radiomics models.

### 3.4. The Comparisons of Different Radiomics Models

**Figure 5:**
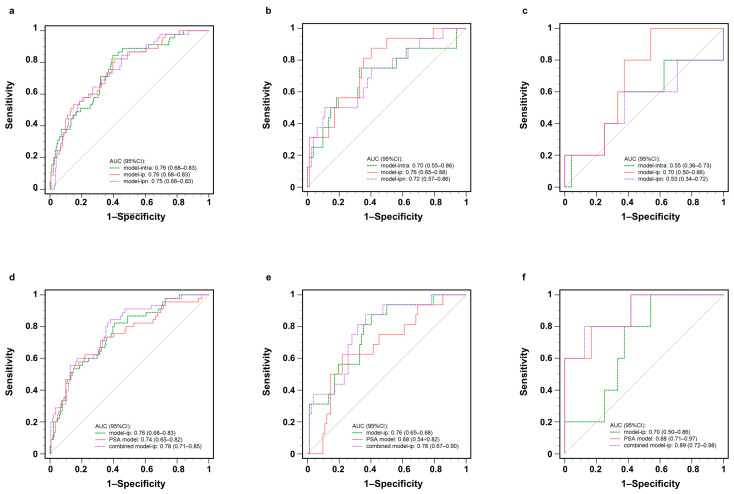
ROC curve analysis of the three best radiomics models in the (**a**) training cohort, (**b**) internal validation cohort, and (**c**) external validation cohort. ROC curve analysis of model-ip, PSA model, and combined model-ip in the (**d**) training cohort, (**e**) internal validation cohort, and (**f**) external validation cohort. ROC: Receiver operating characteristic; PSA: prostate-specific antigen; AUC: area under the curve.

Table 3 compares the differences between the three most superior radiomics models. Their ROC curves are shown in Figure 5. Model-ip and model-ipn were compared against model-intra separately. In all three cohorts, the AUC values of model-ip were higher than those of the other radiomics models, even though there was no statistically significant difference in the Delong test (*p* > 0.05). However, based on NRI and IDI tests, there was a substantial difference (*p* < 0.05) between model-intra and model-ip in the internal cohorts. A slight performance improvement was also observed in the internal validation cohort by the NRI test (*p* < 0.05) when model-intra and model-ipn were compared.

### 3.5. The Comparisons Between Different Combined Models and PSA Model

**Figure 6:**
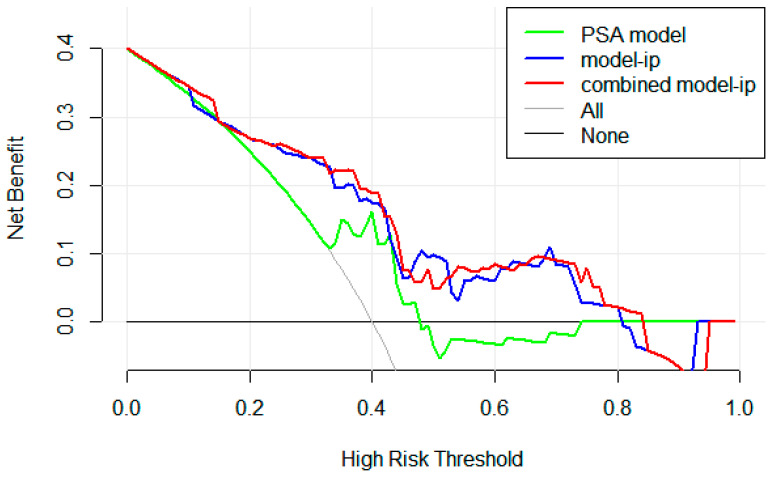
Decision curve analysis for the models.

To test the improvement of the radiomics models to the PSA model, Table 4 shows the difference between the three selected combined models and the corresponding PSA model. There was no significant difference in the Delong test, while in the NRI test, the combined model-ip and the PSA model were not only statistically significant but also had positive benefits in both internal cohorts. In the IDI test, all models in the internal cohorts were statistically significant relative to the PSA model, but the combined model-ip and PSA model had the highest and greater than 0 value, at 0.10 (0.06–0.15) and 0.14 (0.05–0.23), respectively (*p* < 0.05). At the same time, the AUCs of the combined models in the external cohort are not lower than the PSA model. Furthermore, in the IDI test, it can be seen that the model-ip has the most significant improvement effect on the PSA model. Therefore, combining the three methods, we found that model-ip could help the PSA model to predict persistent PSA and provide its optimal performance. Figure 4 shows the ROC curves of model-ip, the PSA model, and the combination of both. As demonstrated in Figure 6, the DCA reveals that within the threshold probability range of 20–80%, the model-ip and combined model-ip yield significantly greater net benefit than the PSA model.

## 4. Discussion

This study demonstrates that both model-ip, incorporating intratumoral and peri-tumoral RFs, and its combined version with serum PSA effectively predict persistent PSA—a well-established indicator of poor short-term prognosis and potential biochemical recurrence following RP. While postoperative PSA monitoring remains the clinical standard for surveillance, practical limitations including extended follow-up intervals and patient compliance gaps may delay critical interventions. Our integrated approach, utilizing preoperative PET-derived RFs extracted from distinct SUVmax zones alongside baseline PSA values, provides enhanced risk stratification to optimize postoperative management strategies.

Among the radiomics models, model-ip, model-ipn, and model-intra exhibited the highest AUC values. Their shared dependency on zone-intra RFs aligns with established studies correlating intratumoral heterogeneity with cancer prognosis [28,29]. Zone-intra represents the core part of the lesion where tumor cell heterogeneity is so obvious that it could potentially drive rapid PSA escalation. To evaluate the incremental prognostic value of extra-tumoral regions, we compared model-ip and model-ipn against model-intra using the DeLong test; however, no significant differences were observed, possibly due to a type II error from a limited sample size. Nevertheless, NRI and IDI analyses confirmed model-ip’s statistical superiority (*p* < 0.05), likely attributable to its synergistic capture of intra- and peri-tumoral RFs. This finding implies that peri-tumoral features reflect TME heterogeneity—a known facilitator of tumor progression [19,20,21,22,30,31]—thereby augmenting prognostic stratification.

The observed performance gap between model-ip and model-ipn suggests that the incorporation of RFs from normal tissue may introduce confounding biological variations, potentially diluting tumor-specific signals. Based on this observation, future studies may precisely delineate tumor lesions to minimize interference from normal tissue, though further verification is needed through large sample and multicenter investigations.

Key RFs driving predictive performance included gray-level size zone matrix (GLSZM) and gray-level co-occurrence matrix (GLCM). GLSZM represents the number of occurrences of common gray-scale regions in the recorded image region in 3D (or 2D) areas, and GLCM describes the arrangement information about the pairs of voxels that follow a certain distribution pattern in the two spaces. These texture features have established roles in characterizing tumor aggressiveness [32,33], further supporting their biological plausibility in our models.

In line with clinical practice, we incorporated preoperative parameters into our analysis, identifying tPSA as an independent predictor of persistent PSA—a finding consistent with established evidence [7].

The optimal combined model-ip (PSA model+ model-ip) significantly outperformed the PSA model alone in predicting persistent PSA (NRI = 0.30, IDI = 0.14; *p* < 0.05 *). This enhancement may stem from radiomics’ capacity to capture subvisual tumor heterogeneity beyond conventional biomarkers. Specifically, intra- and peri-tumoral PET features—predominantly texture markers like GLSZM and GLCM—quantify subtle pathological variations in prostate cancer that evade naked-eye detection [34]. While PSA reflects local tumor burden and biological aggression within the prostate, radiomics deciphers spatial heterogeneity in the tumor microenvironment (TME), synergistically refining risk stratification.

External validation on an independent cohort demonstrated clinically consistent trends favoring model-ip and combined model-ip, though statistical significance was limited by sample size. Specifically, all key metrics showed non-inferior or improved performance versus baselines.

Despite these results, our study has limitations, including a small sample size and its retrospective nature. In addition, due to the lack of a unified standard for the regional division of the prostate, the regional division in this study was mainly based on clinical experience and previous research studies [26,27]. Future research should leverage larger multicenter data, accurately divide subregions, and employ prospective cohorts for validation.

## 5. Conclusions

The RFs from distinct ^18^F-PSMA-1007 PET/CT subregions demonstrate a differential prognostic value for persistent PSA. A radiomics model that encompasses the 20–45% SUVmax and 45–100% SUVmax zones, when combined with the PSA model, markedly enhances predictive accuracy. This combined approach provides a non-invasive tool for personalized prognosis stratification, potentially guiding early intervention in high-risk patients. External validation trends affirm its clinical promise, warranting multicenter trials for translation.

## Figures and Tables

**Table 1:** Comparison of clinical characteristics of patients between the training and validation cohorts.

Characteristics	Center 1 (*n* = 325)		Center 2 (*n* = 29)	*p*
	Training Cohort (*n* = 227)	Internal Validation Cohort (*n* = 98)	External Validation Cohort (*n* = 29)	
Age (years)	68.38 ± 7.03	69.00 ± 6.12	67.72 ± 4.77	0.445
Height (m)	1.68 (1.65–1.70)	1.67 (1.63–1.71)	1.70 (1.67–1.71)	0.121
Weight (kg)	67.00 (60.00–74.00)	67.00 (60.00–75.00)	70.00 (61.50–75.00)	0.665
BMI (kg/m^2^)	23.92 (21.97–25.82)	24.44 (21.74–26.26)	23.94 (22.41–25.95)	0.673
tPSA (ng/mL)	12.15 (7.35–20.30)	10.91 (7.61–23.61)	14.58 (7.74–36.81)	0.683
fPSA (ng/mL)	1.52 (0.86–2.25)	1.80 (1.03–2.25)	1.81 (1.05–2.51)	0.670
Tumor SUVmax	12.30 (8.40–21.20)	13.60 (8.70–23.63)	18.80 (15.40–35.00)	0.001 *
ISUP				0.747
1	10 (4%)	3 (3%)	2 (7%)	
2	75 (33%)	30 (31%)	7 (24%)	
3	85 (37%)	40 (41%)	7 (24%)	
4	24 (11%)	11 (11%)	8 (28%)	
5	33 (15%)	14 (14%)	5 (17%)	

BMI: Body mass index; tPSA: total prostate-specific antigen; fPSA: free prostate-specific antigen; SUV: standardized uptake value; ISUP: International Society of Urological Pathology; * *p* < 0.05.

**Table 2:** The performance of models in predicting persistent PSA probability.

Cohort	Model	AUC (95%CI)	Sensitivity (95%CI)	Specificity (95%CI)
Training cohort	model-intra	0.76 (0.68–0.83)	0.84 (0.71–0.94)	0.60 (0.53–0.68)
	model-peri	0.76 (0.68–0.84)	0.76 (0.61–0.87)	0.73 (0.65–0.79)
	model-norm	0.69 (0.60–0.78)	0.73 (0.58–0.85)	0.60 (0.52–0.67)
	model-ip	0.76 (0.68–0.83)	0.82 (0.68–0.92)	0.59 (0.52–0.67)
	model-ipn	0.75 (0.68–0.83)	0.84 (0.71–0.94)	0.55 (0.47–0.62)
	PSA model	0.74 (0.65–0.82)	0.62 (0.47–0.76)	0.78 (0.71–0.84)
Internal validation cohort	model-intra	0.70 (0.55–0.86)	0.75 (0.48–0.93)	0.67 (0.56–0.77)
	model-peri	0.61 (0.44–0.78)	0.44 (0.20–0.70)	0.83 (0.73–0.90)
	model-norm	0.67 (0.55–0.79)	0.81 (0.54–0.96)	0.59 (0.47–0.69)
	model-ip	0.76 (0.65–0.88)	0.88 (0.62–0.98)	0.60 (0.48–0.70)
	model-ipn	0.72 (0.57–0.86)	0.50 (0.25–0.75)	0.89 (0.80–0.95)
	PSA model	0.68 (0.54–0.82)	0.63 (0.35–0.85)	0.78 (0.68–0.86)
External validation cohort	model-intra	0.55 (0.36–0.73)	0.60 (0.15–0.95)	0.67 (0.45–0.84)
	model-peri	0.61 (0.41–0.78)	0.80 (0.28–1.00)	0.63 (0.41–0.81)
	model-norm	0.59 (0.40–0.77)	0.60 (0.15–0.95)	0.63 (0.41–0.81)
	model-ip	0.70 (0.50–0.86)	1.00 (0.48–1.00)	0.46 (0.26–0.67)
	model-ipn	0.53 (0.34–0.72)	0.60 (0.15–0.95)	0.63 (0.41–0.81)
	PSA model	0.88 (0.71–0.97)	0.80 (0.28–1.00)	0.83 (0.63–0.95)

AUC: Area under the curve; CI: confidence interval.

**Table 3:** Comparing the performance of radiomics models by three indexes.

Cohort	Model	AUC (95%CI)	*p*	NRI (95%CI)	*p*	IDI (95%CI)	*p*
Training cohort	model-intra	Reference					
	model-ip	0.76 (0.68–0.83)	0.975	−0.06 (−0.20–0.09)	0.447	0 (−0.05–0.05)	0.954
	model-ipn	0.75 (0.68–0.83)	0.889	−0.05 (−0.18–0.08)	0.443	−0.03 (−0.07–0.01)	0.156
Internal validation cohort	model-intra	Reference					
	model-ip	0.76 (0.65–0.88)	0.367	0.24 (0.02–0.45)	0.029 *	0.09 (0.02–0.16)	0.012 *
	model-ipn	0.72 (0.57–0.86)	0.871	0.23 (0.01–0.44)	0.040 *	0.06 (−0.01–0.13)	0.087
External validation cohort	model-intra	Reference					
	model-ip	0.70 (0.50–0.86)	0.543	0.45 (−0.49–1.39)	0.349	0 (−0.03–0.03)	0.939
	model-ipn	0.53 (0.34–0.72)	0.959	0.03 (−0.91–0.98)	0.945	0.01 (−0.01–0.04)	0.346

Model-intra is the old model in the comparison between NRI and IDI; * *p* < 0.05.

**Table 4:** Comparing the performance of PSA and combined models by three indexes.

Cohort	Model	AUC (95%CI)	*p*	NRI (95%CI)	*p*	IDI (95%CI)	*p*
Training cohort	PSA model	Reference					
	combined model-intra	0.78 (0.71–0.85)	0.327	0.21 (0.06–0.35)	0.005 *	0.10 (0.05–0.14)	<0.001 *
	combined model-ip	0.78 (0.71–0.85)	0.318	0.16 (0.04–0.28)	<0.001 *	0.10 (0.06–0.15)	<0.001 *
	combined model-ipn	0.79 (0.72–0.86)	0.163	0.13 (0.02–0.24)	0.017 *	0.07 (0.04–0.11)	<0.001 *
Internal validation cohort	PSA model	Reference					
	combined model-intra	0.70 (0.55–0.86)	0.737	0.06 (−0.06–0.19)	0.321	0.05 (0.01–0.10)	0.017 *
	combined model-ip	0.78 (0.67–0.90)	0.161	0.30 (0.07–0.53)	0.011 *	0.14 (0.05–0.23)	0.001 *
	combined model-ipn	0.73 (0.60–0.87)	0.568	0.21 (−0.01–0.43)	0.056	0.10 (0.03–0.17)	0.007 *
External validation cohort	PSA model	Reference					
	combined model-intra	0.88 (0.71–0.97)	1.000	−0.22 (−1.15–0.72)	0.649	0.06 (−0.08–0.19)	0.387
	combined model-ip	0.89 (0.72–0.98)	0.480	−0.13 (−1.07–0.80)	0.781	0.07 (−0.08–0.22)	0.362
	combined model-ipn	0.89 (0.72–0.98)	0.480	−0.22 (−1.15–0.72)	0.649	0.05 (−0.08–0.18)	0.422

The PSA model is the old model in the comparison between NRI and IDI; * *p* < 0.05.

## Data Availability

The data that support the findings of this study are available upon request from the corresponding author.

## Supplementary Materials

The following supporting information can be downloaded at https://www.mdpi.com/article/10.3390/cancers17172807/s1, Table S1: The details of each model.

## Abbreviations

The following abbreviations are used in this manuscript:

AUC	Area under the curve
DCA	Decision curve analysis
GLCM	Gray-level co-occurrence matrix
GLSZM	Gray-level size zone matrix
ICC	Intra-class correlation coefficient
IDI	Integrated discrimination improvement
ISUP	International Society of Urological Pathology
LASSO	Least absolute shrinkage and selection operator
mRMR	Maximum relevance minimum redundancy
NRI	Net reclassification index
PCa	Prostate cancer
PSA	Prostate-specific antigen
PSMA	Prostate-specific membrane antigen
RF	Radiomics feature
ROC	Receiver operating characteristic
ROI	Region of interest
RP	Radical prostatectomy
SUV	Standardized uptake value
TME	Tumor microenvironment
tPSA	Total prostate-specific antigen
